# French-speaking children’s freely produced labels for facial expressions

**DOI:** 10.3389/fpsyg.2014.00555

**Published:** 2014-06-04

**Authors:** Reem Maassarani, Pierre Gosselin, Patricia Montembeault, Mathieu Gagnon

**Affiliations:** School of Psychology, University of OttawaOttawa, ON, Canada

**Keywords:** facial expression, emotion, labeling, children

## Abstract

In this study, we investigated the labeling of facial expressions in French-speaking children. The participants were 137 French-speaking children, between the ages of 5 and 11 years, recruited from three elementary schools in Ottawa, Ontario, Canada. The facial expressions included expressions of happiness, sadness, fear, surprise, anger, and disgust. Participants were shown one facial expression at a time, and asked to say what the stimulus person was feeling. Participants’ responses were coded by two raters who made judgments concerning the specific emotion category in which the responses belonged. 5- and 6-year-olds were quite accurate in labeling facial expressions of happiness, anger, and sadness but far less accurate for facial expressions of fear, surprise, and disgust. An improvement in accuracy as a function of age was found for fear and surprise only. Labeling facial expressions of disgust proved to be very difficult for the children, even for the 11-year-olds. In order to examine the fit between the model proposed by Widen and Russell (2003) and our data, we looked at the number of participants who had the predicted response patterns. Overall, 88.52% of the participants did. Most of the participants used between 3 and 5 labels, with correspondence percentages varying between 80.00% and 100.00%. Our results suggest that the model proposed by Widen and Russell (2003) is not limited to English-speaking children, but also accounts for the sequence of emotion labeling in French-Canadian children.

## INTRODUCTION

Children’s ability to recognize emotions from facial expressions plays an important role in their social adaptation. The information provided in the face allows protagonists involved in a social interaction to assess each other’s internal state, and to adjust their behavior in such a way that individual needs can be fulfilled (Izard, 1991; Ekman, 2003). There is strong evidence that the ability to interpret facial expressions in terms of emotion categories is shown in the preschool years when the recognition task is very simple. Bullock and Russell (1985) presented 2-year-olds with two pictures at the time and asked them to point to the face displaying a specific emotion. They noted that performance was above chance level for happiness, anger, fear, surprise, disgust, and sadness. Preschoolers’ performance has also been found to be quite good for some basic emotions when the recognition task involves a choice of three different facial expressions (Harrigan, 1984; Camras and Allison, 1985; Markham and Adams, 1992; Boyatzis et al., 1993).

Important differences in the developmental pattern of the recognition of the different emotions are also well documented. Facial expressions of happiness, anger, and sadness have generally been found to be recognized at an earlier age than those of fear, anger, and surprise. These differences are quite robust. They were reported when children had to select the expression of a given emotion from an array of different expressions (Tremblay et al., 1987; Markham and Adams, 1992; Gosselin, 1995; Gosselin and Larocque, 2000), when they were shown one expression at a time and asked to select the right emotion name from an array of different names (Zuckerman and Przewuzman, 1979; Gosselin et al., 1995), and when they were presented with one expression at a time and asked to generate an emotion label on their own (Harrigan, 1984; Markham and Adams, 1992; Vicari et al., 2000; Widen and Russell, 2003).

It is important to note that children’s performance in recognizing facial expressions varies according to the type of task. Researchers who used the labeling task have generally reported a level of performance lower than those who used the choice-from-an-array task (Harrigan, 1984; Markham and Adams, 1992). Two main interpretations have been proposed to explain this difference. One possibility is that the lower-level performance in the labeling task results from a problem of accessibility of emotion labels. While emotion labels are easily accessible in the choice-from-array task, children have to retrieve them from their long term memory when they perform the labeling task. A second possibility, proposed by Russell (1994), is that the better performance in the choice-from-array task results from a method artifact. When presented with a target facial expression and several labels, children might choose the right label not because they know what emotion is portrayed by the target facial expression, but because they have eliminated the other labels. According to this explanation, children use their knowledge of the facial expressions they know the best to eliminate response options until they are left with only one option. According to this view, the processing of information involved in the choice-from-array task is not representative of the type of processing that takes place in everyday life. When children interact with other people, they see their faces and they have to retrieve the appropriate emotion concept from their long term memory. They are not provided with various labels as is the case in the choice-from-array task.

Recently, Widen and Russell (2003, 2008, 2013) proposed a model accounting for the age changes in the labeling of facial expressions. In a series of studies, these authors examined the number of labels used by children in free-labeling tasks. One facial expression was presented at a time, and children were asked to say what the stimulus person was feeling. Their analysis led them to propose a model of progressive differentiation between the emotion labels accounting for 81.3% of the children’s responses. According to this model, children begin with a very simple meaning system for interpreting facial expressions. When children use only one word to label facial expressions, this word most often refers to happiness. When they use two words, at the age of 40 months on average, these words most often refer to happiness and sadness, or to happiness and anger. At the following level, children use three words: happy, angry, and sad. Surprise and fear are added at the age of 56 months, on average, with some children adding fear to happy, angry, and sad while others add surprise to these three words. At the next level (at 62 months on average), children use five labels: happy, sad, angry, scary, and surprise. At the last level, children add disgust to the lexicon they use to label facial expressions. The last level is achieved by children at the age of 66 months, on average.

The differentiation model thus posits developmental changes in the number and in the size of emotion categories. Some emotion categories, like surprise or disgust, are never used (or rarely used) by 3-year-olds but are used quite often by older children. Other categories, like happiness, sadness, and anger, are used to describe the appropriate facial expression but also other irrelevant facial expressions. As children get older, they tend to use these categories to describe these other irrelevant facial expressions less often. Developmental changes thus affect the size of emotion categories. At a given age, some emotion categories are very broad and include the relevant facial expression as well as irrelevant facial expressions. Other emotion concepts are rarely used (their labels are rarely produced), even when the relevant facial expression is shown to the child.

According to Widen and Russell (2013), the age changes in the labeling of facial expressions reflect the process of concept formation. The concept of anger, for instance, corresponds to the relation between several components such as children’s knowledge of the causes of anger, of its behavioral consequences, of its facial, vocal, and behavioral correlates, of its bodily changes, and of the words used to name it. The emotion concepts do not emerge fully formed, but develop gradually, one at a time. Furthermore, the order of addition of the components is not necessarily the same for all emotions. The authors assert that facial expressions may be added to the concept of happiness earlier than for the concepts of fear or disgust. The age at which a component is added to a given emotion concept is thought to depend upon the regularity of a particular cue. If facial correlates of a given emotion are highly variable, they are less likely to be added to the concept than if they are a little variable. Culture and language are also expected to exert an effect on the particular time components are added to emotion concepts. Social sanctions for expressing certain emotions might delay the time at which their facial correlates are added to the corresponding emotion concepts. The extent to which cultures emphasize some emotions compared to others is likely to facilitate the addition of emotion words to the corresponding emotion concepts. Emotions that are often discussed by parents, educators, or other people interacting with children are more likely to be named appropriately than those that are not. Current evidence indicates that languages partition the emotion domain differently (Shweder et al., 2008). To the extent that some languages make clearer distinctions between emotions than other languages do, one can expect children speaking these languages to better label emotions or to label them sooner.

The model proposed by Widen and Russell (2003, 2008, 2013) is based on data gathered in studies using free-labeling tasks with English-speaking children. It is not known whether the model could account for the recognition of facial expressions of children who use languages other than English. According to these authors, the process by which children learn to label facial expressions is affected by the way the culture emphasizes particular concepts. For instance, they suggested that the slow pace at which children improve in producing the correct label for disgust might reflect the fact that this emotion is not emphasized in North-American culture. They also proposed that the differentiation between emotion categories might be affected by the lexicon associated with a given language. Specifically, they hypothesized that the label *dégoûté* in French might be used earlier by French-speaking children than the corresponding label in English (*disgusted*) because its meaning is simpler: it is more closely associated with bad tastes and food. In English, the term *disgusted* also refers to morality and persons as well as food.

The general aim of this study was to investigate the free-labeling of facial expressions in a sample of French-Canadian children. We were especially interested in the developmental changes that took place during the second half of childhood (5–11 years) because it is not yet clear at what age children are able to succeed in the labeling of facial expressions of fear, surprise, and disgust. First, we examined whether the sequence of use of emotion categories in this population was similar to the sequence identified by Widen and Russell (2003). In order to do so, the labels produced by the children were coded by independent judges to determine the emotion category to which they referred. Our coding procedure was very similar to that used by Widen and Russell (2003), but adapted to the French language. We hypothesized that if a child used only one emotion category while performing the task, that category would refer to happiness. If a child used only two different emotion categories to describe the different facial expressions, these categories would refer to happiness and sadness or to happiness and anger. If a child used three different emotion categories, they would refer to happiness, sadness, and anger. To the extent that four different emotion categories were used, they would refer to happiness, sadness, anger, and fear or to happiness, sadness, anger, and surprise. If five different categories were used, they would refer to happiness, sadness, anger, fear, and surprise.

Second, we examined the changes in accuracy in labeling facial expressions. Based on the findings of previous studies (Harrigan, 1984; Markham and Adams, 1992; Vicari et al., 2000; Widen and Russell, 2008), we expected labeling accuracy for fear, surprise, and disgust expressions to improve between the ages of 5 and 11.

Third, we examined the number of emotion categories used by school-age children. According to Widen and Russell (2003, 2008), facial expressions that are put in the same category in early childhood (like fear and surprise or disgust and anger) are later put in different categories. Thus, we expected the number of categories used by children to label facial expressions to increase between the ages of 5 and 11.

Fourth, we were interested in developmental changes regarding the size of emotion categories, the latter being defined as the number of different facial expressions put in that category. According to the differentiation model, the size of some emotion categories increases as a function of age while the size of other categories decreases. Specifically, we expected the size of the categories referring to happiness, sadness, and anger would decrease while the size of the categories referring to fear, surprise, and disgust would increase between the ages of 5 and 11. According to the differentiation model, the categories referring to happiness, sadness, and anger have already appeared at the age of 5, and they narrow during the following years as they are better differentiated from the other categories. In contrast, the categories referring to fear, surprise, and disgust appear later, meaning that young children are less likely than older children to use these labels when presented with facial expressions depicting these emotions.

Fifth, we investigated the spectrum of the words used by school-age children to label facial expressions. According to parental reports, most English-speaking children begin to use words referring to happiness, sadness, and anger between the age of 2 and 3 (Ridgeway et al., 1985). Analyzing spontaneous speech in young children, Wellman et al. (1995) provided more direct evidence of this. They found that 2-year-olds use several words, like happy, sad, and mad, to refer to the emotions they experience as well as those experienced by other people. Dunn et al. (1987) and MacWhinney (2000) also examined spontaneous conversations in young English-speaking children and report that most 3-year-olds use words referring to disgust (like disgust, yuck) in spontaneous conversation, although they do so infrequently. To our knowledge, such an analysis of children’s lexicon has not yet been conducted in French-speaking children. This analysis was informative with respect to richness of children’s lexicon in the emotion domain. It allowed us to determine whether the labels used by emotion researchers (Sander and Scherer, 2009) to name the basic emotions (such as happiness, fear, anger, surprise, sadness, and disgust) are also used by children when they have to label facial expressions. It also allowed us to identify the labels that are used the most often by school-age children. The results we gathered with respect to the latter issue have implications for designing recognition tasks in future studies.

## MATERIALS AND METHODS

### PARTICIPANTS

The participants in this study were 137 children recruited from three middle-class elementary schools located in Ottawa, Ontario, Canada. The ages of these children ranged from 5 to 11 years old. The children were divided into seven different age groups: 17 (8 girls) were 5 years old (*M* = 5.57, SD = 0.27), 21 (9 girls) were 6 years old (*M* = 6.48, SD = 0.31), 21 (12 girls) were 7 years old (*M* = 7.44, SD = 0.28), 15 (10 girls) were 8 years old (*M* = 8.33, SD = 0.24), 17 (7 girls) were 9 years old (*M* = 9.48, SD = 0.32), 25 (15 girls) were 10 years old (*M* = 10.47, SD = 0.31), and 20 (9 girls) were 11 years old (*M* = 11.80, SD = 0.32). All participants had French as their mother tongue (no one had English as their mother tongue) and were educated in French. Only children with parental consent took part in the study. The recruitment and the treatment of participants were carried out in conformity with the ethical standards for research at the University of Ottawa.

### MATERIAL

The materials shown to participants included six drawings of animals and 14 pictures of facial expressions. The presentation of animals served to verify that children were able and willing to provide labels. The drawings of animals (cat, dog, cow, rabbit, horse, and chicken) were taken from the *Animal Category *of Clipart, Microsoft Office, and were modified with Photoshop in order to enlarge their original format. The facial expressions were identical to those used by Widen and Russell (2003, Study 3) and included seven facial expressions (neutral, happy, sad, mad, scared, surprised, and disgusted) made by a school-age boy and a school-age girl. The pictures were taken from the Linda Camras collection and were coded with the FACS (Ekman and Friesen, 1978) to ensure that they were representative of prototypical facial expressions. Camras et al. (1983) presented evidence that these facial expressions were well recognized by adults.

### PROCEDURE

The study was conducted with each participant individually, in an area located close to his or her classroom. The experiment involved one 15-min period and included two tasks: the labeling of animals, always performed first, and the labeling of facial expressions. The drawings of animals were presented in a random order, one at a time, until the participant provided his or her response. The facial expressions were presented in a similar way, except that all of the expressions for a given model were presented in a row. First the experimenter showed the neutral face of the model and indicated his or her name (Marc or Suzanne), and then the six emotional expressions in a random order. The order of the models (boy first or girl first) was balanced across participants. If participants were not able to label a facial expression, they were asked if they had ever seen someone making the same expression, and how that person was feeling at that moment. If the participants could still not answer, the experimenter asked them if they had made the same face, and how they felt at that moment. The participants’ responses were written down by the experimenter.

### SCORING

Participants’ responses were coded separately by two judges (undergraduate students) according to a coding scheme prepared by two other judges (graduate students). The coding scheme (see **Table 1**) included words, and expressions related to the six basic emotions and found in several French dictionaries. Each judge coded all the labels produced by the 137 participants and was unaware of the participants’ age and gender as well as the type of expression that were presented. The inter-rater agreement was 0.90 κ and disagreements between the judges were later resolved by discussion. These disagreements always occurred for non-expected words produced by the participants.

**Table 1:** Scoring scheme used to classify children’s labels into emotion categories.

Category	Labels associated with the category
Happiness	Content, heureux, joyeux, réjoui, ravi, jovial, gai, satisfait, épanoui, enthousiaste, se sent bien
Fear	Appeuré, a peur, effrayé, paniqué, terrorisé, inquiété, craintif, affolé, alarmé, angoissé
Anger	Fâché, colérique, en colère, offusqué, contrarié, agacé, exaspéré, irrité, indigné, révolté, contrarié, se sent méchant, frustré
Surprise	Surpris, étonné, ahuri, stupéfait, ébahi, effaré, impressionné
Sadness	Triste, malheureux, peiné, attristé, morose, chagriné, maussade, accablé, abattu, déprimé, déçu
Disgust	Dégoûté, dégueulasse, dégueu, écoeuré, répugné, se sent ouache
Other	Other words or expressions not included in the previous lists
Don’t know	No response

In the context of this study, the size of an emotion category refers to the number of different expressions put in it. For example, if a child used the label sadness at least once when presented with sadness and anger expressions but never used it when presented with the other expressions, we considered that the size of his or her category of sadness was 2. If a child used the label surprise at least once when shown expressions of surprise, happiness or fear, the size of his or her surprise category was scored as 3. Note that perfect labeling of facial expressions entails a size of 1 for each emotion category, meaning that a given category is used only for the corresponding facial expression and never for other facial expressions.

## RESULTS

Nearly all the participants (135 out of 137) completed both tasks. Two female participants (a 5-year-old and a 10-year-old) failed to provide labels for facial expressions for most of the trials. The data associated with these participants were not considered in the following analyses.

### CHILDREN’S ABILITY TO PRODUCE VERBAL LABELS FOR ANIMALS

Before performing the main task, children were asked to label animals. This first part of the procedure was intended to verify that children were able and willing to provide labels. Mean accuracy for labeling animals was 92.59% (SD = 14.26) for the 5-year-olds, 94.44% (SD = 10.97) for the 6-year-olds, 96.83% (SD = 6.71) for the 7-year-olds, and between 99.00% and 100.00% for the four older groups. These results are in agreement with those reported by Widen and Russell (2003) and provide clear evidence that even the 5-year-olds understood the task and were able to produce labels.

### THE SEQUENCE OF USE OF EMOTION CATEGORIES

In order to examine whether the model proposed by Widen and Russell (2003) accounted for the responses produced by the participants of this study, we looked at the number of participants who had the predicted patterns of responses. For example, if a given participant produced only two different labels while performing the task, these labels had to refer to sadness and happiness or to anger and happiness. Otherwise, the participant was considered an instance of non-correspondence. If a given participant produced three different verbal labels, these labels had to refer to happiness, anger, and sadness. The number of participants who had a response pattern fitting with the model is presented in **Table 2**. The overall percentage of correspondence was 88.52%, with values ranging from 80% to 100% depending on the number of categories that were used. Given the age range of the participants, we expected them to use several emotion categories. As shown in **Table 2**, 70 children (51.85%) used five categories, 35 (25.93%) four categories, and 15 (11.11%) three categories. Surprisingly, very few children (13 out of 135) used the six categories.

**Table 2:** Correspondence between the emotion categories used by the participants and the sequence proposed by Widen and Russell (2003).

Nb of categories	Nb of matches	Nb of non-matches	Percentage of matches
1	0	0	na
2	2	0	100.00
3	12	3	80.00
4	28	7	80.00
5	66	4	94.3
6	na	na	na
Overall	108	14	88.52

### CHANGES IN THE ACCURACY OF THE LABELING

Mean accuracy in the labeling task was rather low in the 5-year-olds (50.98%), and improved only gradually over the next 6 years, reaching 62.30%, 66.27%, 71.11%, 72.55%, 77.43%, and 75.83% in the older groups, respectively. As the data were not normally distributed, nor amenable to a normal distribution even after various types of transformations, we used the Kruskall–Wallis test to assess the effect of age on overall performance. The test indicated a significant effect of age, χ^2^ (df = 6, *N* = 135) = 39.50, *p *< 0.0001. Dunn’s multiple comparison test for independent samples showed that mean accuracy for the 5-year-olds was lower than for the 8-year-olds and older children, and lower for the 6- and 7-year-olds than for the 10-year-olds and older groups.

**FIGURE 1:**
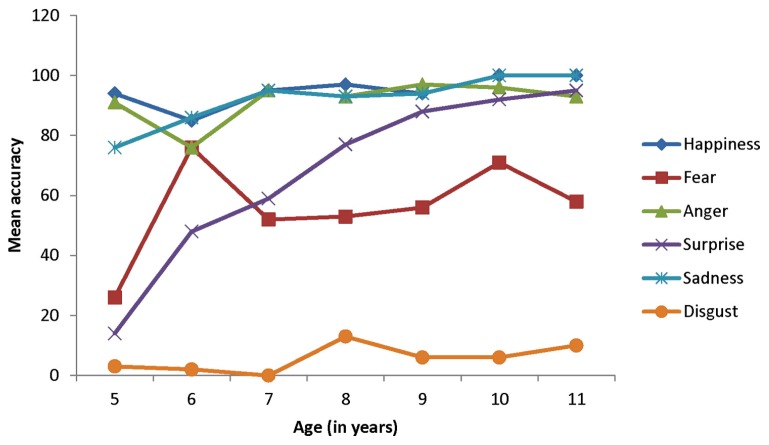
**Mean accuracy (expressed in percentage) in labeling facial expressions as a function of age and type of expression**.

In order to get a more specific picture of the performance in the task, we then turned to the labeling of each type of expressions. As the task included two trials per type of expression, the participants could be credited with 0, 1, or 2 points. **Figure 1** illustrates participants’ mean accuracy in terms of percentages. As one can see from this figure, mean performance was high in the 5-year-olds and in the older groups for happiness, anger, and sadness expressions. However, the situation was different for the three other types of expressions. Mean accuracy was very low in the 5-year-olds for fear and surprise expressions, with major improvement over the following years.

Given that the data were not normally distributed, nor amenable to a normal distribution, the Kruskall–Wallis test was used to examine age differences between the age groups. The test indicated a significant effect of age for fear, χ^2^ (df = 6, *N* = 135) = 19.04, *p *< 0.004, and surprise expressions, χ^2^ (df = 6, *N* = 135) = 45.59, *p *< 0.0001. Dunn’s multiple comparison test for independent samples showed that mean accuracy in labeling fear was lower for the 5-year-olds than for older groups. However, no other differences were significant. The test also showed that mean accuracy in labeling surprise expressions was higher for the 9-year-olds and older children than for the 5-year-olds, and higher for the 9-year-olds and older children than for the 6-year-olds.

**FIGURE 2:**
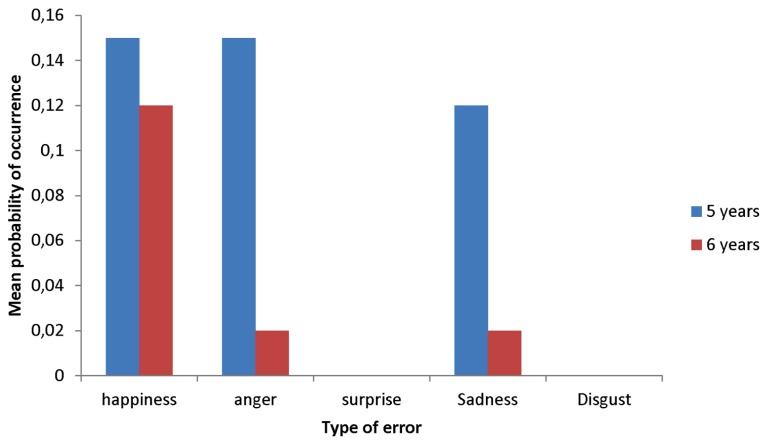
**Mean probability of occurrence of errors for fear expressions as a function of type of error**.

**FIGURE 3:**
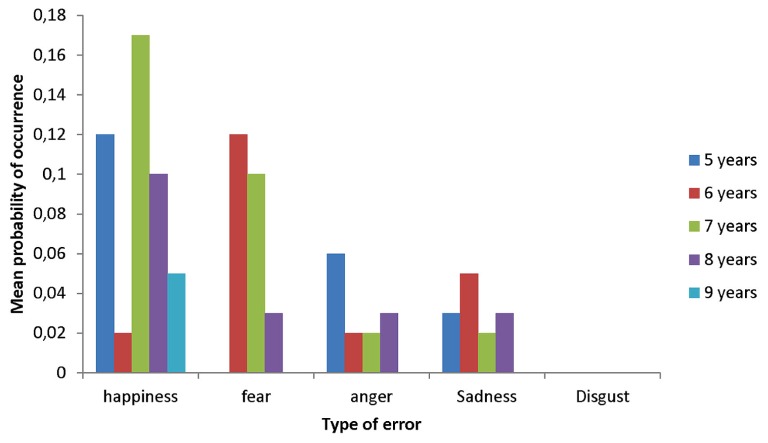
**Mean probability of occurrence of errors for surprise expressions as a function of type of error**.

In order to better understand the improvement in labeling accuracy for fear and surprise, we examined the frequency of the errors. We were specifically interested in knowing which other irrelevant emotion categories they used when shown these two types of expressions. When the 5-year-olds were presented with fear expressions, their errors consisted in interpreting them as happiness, anger, and sadness (see **Figure 2**). The Wilcoxon test for independent samples indicated that the 6-year-olds were less likely than the 5-year-olds to interpret fear expressions as anger, χ^2^ (df = 1, *N* = 38) = 375.50, *p *< 0.04. However, no significant differences were found for the two other types of errors. As shown in **Figure 3**, the 5-year-olds tended to interpret surprise expressions as happiness, the 6-year-olds as fear, and the 7-year-olds as happiness and fear. The Kruskall–Wallis test did not reveal any significant changes related to age in the frequency of these two types of errors.

As we mentioned earlier, disgust expressions were generally poorly labeled by the children between the ages of 5 and 11. As we did not find any improvement in the labeling accuracy as a function of age, we examined the frequency of the errors for the participants pooled together. The Friedman test for related samples indicated significant differences in the probability of the different types of errors, χ^2^ (df = 4, *N* = 135) = 225.27, *p *< 0.0001. Interpreting disgust expressions as anger (*M* = 0.25) was far more common than interpreting them as happiness (*M* = 0.01), fear (*M* = 0.00), surprise (*M* = 0.00), or sadness (*M* = 0.02).

### CHANGES IN THE NUMBER OF EMOTION CATEGORIES USED BY THE CHILDREN

The mean number of emotion categories used by the participants is presented in **Table 3**. The Kruskall–Wallis test indicated a significant effect of age, χ^2^ (dl = 6, *N* = 135) = 38.77, *p *< 0.0001. Dunn’s multiple comparison test revealed that the 8-year-olds and older groups used more categories than the 5-year-olds. No other significant differences were found between the groups.

**Table 3:** Number of categories used by the participants.

Age	*M*	SD
5	3.47	0.87
6	4.19	0.87
7	4.48	0.75
8	4.73	0.80
9	4.88	0.60
10	5.00	0.51
11	4.90	0.55

### CHANGES IN THE SIZE OF EMOTION CATEGORIES

**FIGURE 4:**
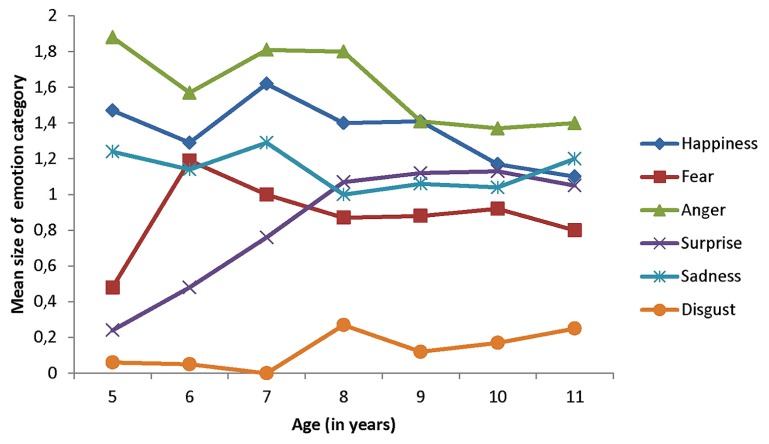
**Mean size of emotion categories as of function of age**.

The mean size of each emotion category is presented as a function of age in **Figure 4**. Age effects in the size of emotion categories were detected with the Kruskall–Walls test for fear, χ^2^ (dl = 6, *N* = 135) = 16.39, *p *< 0.01, and surprise χ^2^ (dl = 6, *N* = 135) = 39.90, *p *< 0.0001. The Dunn multiple comparison test showed that the size of the fear category was smaller in the 5-year-olds than in the older groups, but failed to detect any other differences. This test also indicated that the size of the surprise category was smaller in the 5- and 6-year-olds than in the 8-year-olds and older groups. Interestingly, the size of the disgust category was very small in the 5-year-olds (*M* = 0.06) and still small in the 11-year-olds (*M* = 0.25).

### FREQUENCY OF THE LABELS PRODUCED BY THE CHILDREN

In the previous sections, we were concerned with the use of emotion categories. The labels produced by the children were categorized in different emotion categories according to our coding scheme. We now turn to the specific labels produced by the participants in order to document the emotional vocabulary in later childhood. **Table 4** indicates the total number of times a given word was used for all the children pooled together. Note that this table includes all the words used by children, whether they were correct or not. This is why the number of terms in **Table 4** is different from that in **Table 1**. Of the 1105 labels produced by the children, 255 referred to happiness, 233 to sadness, 319 to anger, 129 to fear, and 154 to surprise. Labels referring to disgust were produced only 15 times, which is very low given the number of participants (135) and the number of trials in the task (12).

**Table 4:** Frequency of the labels used by the participants for the overall task.

Happiness	Content	209	81.96
	Heureux	16	6.27
	Joyeux	7	2.75
	Bien	19	7.45
	Souriant	3	1.18
	Trouve ça drôle	1	0.39
	Total	255	100.00

Disgust	Dégoûté	9	60.00
	Dégueu (lasse)	4	26.66
	Aark	1	6.67
	Dégusté	1	6.67
	Total	15	100.00

Sadness	Déçu	2	0.86
	A de la peine	6	2.58
	Triste	224	96.14
	Malheureux	1	0.42
	Total	233	100.00

Anger	Choqué	7	2.20
	En colère	2	0.63
	Frustré	4	1.26
	Fâché	296	93.08
	Méchant	5	1.57
	Furieux	1	0.31
	Enragé	2	0.63
	En crisse	1	0.31
	Total	319	100.00

Fear	Appeuré, épeuré, appeurant	15	11.63
	A peur	100	77.52
	Inquiet	4	3.10
	Panique	2	1.55
	Peureux	3	2.32
	Effrayant	2	1.55
	Effrayé	2	1.55
	Menacé	1	0.78
	Total	129	100.00

Surprise	Surpris	141	91.56
	Étonné	7	4.54
	Impressionné	3	1.95
	Épaté	3	1.95
	Total	154	100.00

As one can see from **Table 4**, the number of different labels varied between 4 and 8 per emotion category. It is clear from this table that some labels were produced far more often than others. The label *content* represented 81.96% of all the labels associated with happiness, *triste* 96.14% of all those associated with sadness, *fâché* 93.08% of all of those associated with anger, and *surpris* 91.56% of all of those associated with surprise. The most common label associated with fear was the expression *a peur* (77.52%), followed by three words that we grouped together (11.63%) because they shared the same root (*peur*). Of these three words, only *apeuré* was appropriate in the context of the task because it refers to the state of the stimulus person. The words *épeurant* and *apeurant* are not correct because they refer to the effect produced by the face of the stimulus person on other people.

As we mentioned before the labels referring to disgust were used only 15 times. Among the four different words that were used, *dégoûté* was the most frequent, with a relative frequency of 60.00%, following by *dégueu* and *dégeulasse* (26.66%) which are familiar expressions.

## DISCUSSION

The main objective of this study was to investigate whether the model proposed by Widen and Russell (2003) could account for the labeling of facial expressions in French-Canadian children. Our examination of this question included analyses concerned with the sequence of use of emotion categories, the accuracy of labeling, the number of emotion categories used by the children, and the size of emotion categories.

We first hypothesized that the sequence of use of emotional categories by French-Canadian children would correspond to the sequence proposed by Widen and Russell (2003). Our results strongly support this hypothesis. From the 122 children who used between 2 and 5 categories, 88.52% had a response pattern fitting the model, a value slightly higher than the value (81.30%) reported by Widen and Russell (2003) for children between 2 and 6 years of age. The fact that the children who took part in this study were Canadian might explain in part the strong fit we observed between our data and the proposed model. One could argue that the cultural differences regarding the socialization of emotions between English-American and French-Canadian children are perhaps not strong enough to exert an effect on children’s understanding of emotions, in particular, on the way they interpret facial expressions. However, our participants were different from those examined by Widen and Russell (2003) on the linguistic level. The fact that their model accounted for the response patterns of French-speaking children suggests that it is more generalizable than what was once thought. Of course, additional research is needed to determine whether the model could account for the labeling made by other French-speaking populations, such as those in France, Belgium, and some African countries.

According to Widen and Russell (2003, 2013), emotion concepts emerge gradually, with their different prototypical components (causes, behavioral consequences, facial expressions, emotion words, etc.) being added one at a time and not necessarily in the same order for all emotions. In the case of disgust, for example, Widen and Russell (2013) found that this emotion is first conceptualized as a feeling of unhappiness. Later, the prototypical cause is added to the concept, followed by the label, then the prototypical behavioral consequences, and finally the prototypical facial expression. As children acquire more knowledge about the different prototypical components and become able to link them together in the proper temporal order, they gradually develop a more differentiated script for each basic emotion. This model holds that children abstract the prototypical components from the various events they observe, meaning that they sort specific experiences into general rules or classes. The prototypical cause of sadness, for example, may be losing a valued object, its prototypical subjective feeling may be a feeling of emptiness, and the prototypical behavioral consequence may be social withdrawal.

The labeling of facial expressions is illustrative of one part of this process, with some facial expressions being linked with emotion words earlier than other facial expressions. The results we obtained in this study generally support the developmental pattern predicted by the model. As expected, we found evidence of improvement in accuracy for fear and surprise expressions, meaning that children improve their ability to link the prototypical fear and surprise expression with words referring to fear and surprise. We did not observe improvement in accuracy for disgust expressions, but, in agreement with the model, performance for this type of expression was much worse than for the other expressions.

Our analyses allowed us to identify when the improvement in labeling took place in later childhood. Fear and surprise expressions were found to have different developmental patterns. While the improvement in accuracy took place between the ages of 5 and 6 for fear expressions, it was more gradual for surprise expressions. Our analyses also allowed us to specify the type of differentiation accounting for the improvement in accuracy for fear expressions. We found that 6-year-olds were less likely than 5-year-olds to interpret them as anger.

The fact that we did not detect an improvement in accuracy for disgust expressions is surprising. As shown in **Figure 1**, accuracy for this type of expression was very low in the 5-year-olds and remained very low for the next 6 years. Contrary to the suggestion offered by Widen and Russell (2003), French-speaking children, at least French-Canadian children, do not seem to do better than English-American children. If, as they speculated, the word *dégoût* is less equivocal than the word *disgust* because it refers primarily to bad food and tastes, this semantic difference does not seem to have favored the participants of our study. The fact that children tended to interpret disgust expressions as portraying anger is in agreement with recent research carried out by Widen and Russell (2008, 2010) on the labeling of disgust expressions. Furthermore, this observation is not limited to the particular set of photographs we used, as Widen and Russell (2003) presented their participants with different sets of photographs.

What could account for the low performance in the labeling of disgust expressions? According to the differentiation model, children abstract the relations between prototypical facial expressions and emotion labels from the various events they observe. One possibility would be that children are rarely exposed to words referring to disgust, limiting in this way their opportunities to abstract the relation between these words and the prototypical disgust expression. Estimates of word frequency in American English, as indexed by the CHILDES database (MacWhinney, 2000), indicate that children are less exposed to words referring to disgust than those referring to happiness, fear, anger, surprise, or sadness. There are currently no comparable estimates for French Canadian children, but the Manulex database (Lété et al., 2004) also suggests that words referring to disgust are much less common than those referring to other emotions in textbooks intended for French children. A second possibility would be that children have more difficulty abstracting the prototypical disgust facial expression. However, current evidence does not provide support for the fact that children lack the perceptual ability to discriminate between disgust expressions and other expressions. Gagnon et al. (2010) presented school-age children with pairs of facial expressions and asked them to say which of the two elements matched a target facial expression. Performance for trials involving the disgust face as the target expression was found to be very good, even in the 5- and 6-year-olds, and improved only slightly between the ages of 5 and 10.

The differentiation process postulated by Widen and Russell (2003, 2013) involves two types of changes in emotion categories. As a result of concept formation, children are expected to use a greater number of emotion categories when they label facial expressions, and the size of their emotion categories (indexed by the number of different facial expressions put in a given category) is expected to change over childhood. Categories that were the first to emerge in childhood are supposed to narrow as a function of age, while those that appeared later are supposed to increase. Our results partially supported these contentions. We found that the 8-year-olds and older groups used more categories than the 5-year-olds (see **Table 3**) and age differences for the size of the categories fear and surprise were in the expected direction. Specifically, the category fear was bigger in the 6-year-olds and older groups than in the 5-year-olds, and the category surprise was bigger in the 8-year-olds and older groups than in the 5- and 6-year-olds (see **Figure 4**). Interestingly the mean size of both categories was substantially lower than 1 in the 5-year-olds, meaning that children failed to use these categories when presented with facial expressions of fear and surprise.

We did not detect any increase in the size of the category disgust as a function of age. Again, this observation was unexpected considering the fact that our sample of participants included children older than those of Widen and Russell’s (2003) study. The mean size of the category disgust was substantially below 1, meaning that children rarely put the disgust expression in the proper category. Recent evidence gathered by Widen and Russell (2008, 2010) also suggests that the labeling of the disgust facial expression has a very slow developmental pattern.

One original contribution of this study was to document the children’s lexicon in the labeling of facial expressions. We identified a total of 33 different words produced by children when they performed the task. Only four different words or expressions were produced in reference to sadness, surprise, and disgust. The variety of terms was somewhat greater for happiness, anger, and surprise, with values ranging between 6 and 8.

One striking feature of **Table 4** is the high relative frequency of six words or verbal expressions. The words *content, triste, fâché, and surpris* accounted for 82, 96, 93, and 92%, respectively, of all the words referring to the categories happiness, sadness, anger, and surprise. As for the expression *a peur*, it accounted for 78% of all the words produced in relation to fear. As we mentioned earlier, few words referring to disgust were produced by the children. The word *dégoûté*, along with its familiar form (*dégeu* and *dégueulasse*) accounted for 86.66% of them (13 out of 15). It is interesting to note that the six words or verbal expressions most used by the children are the same as those used by emotion theorists in the French literature (Sander and Scherer, 2009).

The high relative frequency of these six words or verbal expressions has implications for designing recognition tasks. Past research has used two variants of the choice-from-array task. In one variant, participants were presented with one verbal label at a time, and asked to point to the appropriate facial expression among an array of facial expressions. In the second variant, the participants were presented with one facial expression at a time, and asked to choose the appropriate label among an array of labels. In both instances, researchers interested in the recognition of facial expressions in French-speaking children could benefit from presenting their participants with the six words or verbal expressions that our participants used the most frequently.

We mentioned earlier that the slow developmental pattern of the labeling of disgust facial expressions could result from the fact that disgust is not emphasized by North-American culture to the same extent as other emotions, making labels referring to disgust less accessible for children. The available databases concerned with the frequency of emotion words in written and audio media provide some support for this hypothesis, but additional work is required to determine whether this explanation is valid for French-Canadian children. As we said earlier, no evidence is currently available with respect to the frequency of emotion words in this population.

Fear and surprise expressions were also found to have slower developmental patterns than those of happiness, sadness, and anger expressions. However, exposure to emotion words does not seem to account for these differences as much as is the case of disgust. Estimates of word frequency, as indexed by the CHILDES (MacWhinney, 2000) and Manulex databases (Lété et al., 2004) suggest than English-American and French children are not less exposed to words referring to fear and surprise than those referring to sadness and anger.

What other factors could then explain the slow developmental patterns in the labeling of fear and surprise expressions? One possibility could be that some emotions tend to follow each other in a short period of time. For example, many situations conducive of happiness are unexpected, meaning that people experience happiness immediately after having experienced surprise. This is the case when people receive unexpected good news or an unexpected gift. Several situations conducive of fear are also unexpected. The temporal proximity between surprise and these two emotions might make it more difficult for children to abstract the relation between the surprise expressions and other components of the concept. Our results provide some support for this hypothesis, as the most common errors consisted in labeling surprise expressions as happiness and fear. The reason why children had difficulty in labeling fear expressions is not clear. The most common errors made by the 5-year-olds consisted in interpreting these expressions as happiness, anger, and sadness (see **Figure 2**). Performance in the 6-year-olds was better, but they also tended to interpret fear expressions as happiness. This type of error is surprising given that the two emotions differ in terms of valence. This intriguing finding deserves more attention as its robustness needs to be more firmly established by future research.

One limitation of this study concerns the fact that the participants were French-speaking children living in a city where English is the dominant language. Although our participants had French as their mother tongue, and were educated in French, it is possible that their understanding of emotion was influenced by exposure to English. However, one would expect children living in areas where English is the dominant language to use English words when performing the labeling task from time to time. None of the 1105 words collectively produced by the participants were English words. The fact that the children did not say words in English does not necessarily mean that learning this language has not played a role in their categorization of emotions. It is possible that they stopped themselves from switching from French to English. We can only say that we did not find evidence of this when the children performed the task.

The results of this study indicate that the differentiation model proposed by Widen and Russell (2003, 2013) is not limited to English-American children but also accounts for the labeling of facial expressions in French-Canadian children. Although French and English do not partition the emotion domain in exactly the same way, these differences do not seem great enough to exert an effect on the labeling of facial expressions. The same can be said of the influence of the socialization of emotions in children. Although, from a constructivist perspective, culture is expected to shape emotion concepts, the differences between the English-American and French-Canadian cultures regarding the socialization of emotions do not appear to be important enough to affect the labeling of facial expressions. In order to better assess the generality of the differentiation model, future research should focus on populations that differ from the English-American population to a greater extent than what French-Canadians do, both in terms of culture and language.

## Conflict of Interest Statement

The authors declare that the research was conducted in the absence of any commercial or financial relationships that could be construed as a potential conflict of interest.

## References

[B1] Boyatzis C. J. Chazan E. Ting C. Z. (1993). Preschool children’s decoding of facial emotions. *J. Genet. Psychol.* 154 375–382 10.1080/00221325.1993.10532190 8245911

[B2] Bullock M. Russell J. A. (1985). Further evidence on preschoolers’ interpretation of facial expressions. *Int. J. Behav. Dev.* 8 15–38 10.1177/016502548500800103

[B3] Camras L. Allison K. (1985). Children’s understanding of emotional facial expressions and verbal labels. *J. Nonverbal Behav.* 9 84–94 10.1007/BF00987140

[B4] Camras L. Grow G. Ribordy S. (1983). Recognition of emotional expressions by abused children. *J. Clin. Child Psychol.* 12 325–328 10.1080/15374418309533152

[B5] Dunn J. Bretherton I. Munn P. (1987). Conversations about feeling states between mothers and their young children. *Dev. Psychol.* 23 132–139 10.1037/0012-1649.23.1.132

[B6] Ekman P. (2003). *Emotions Revealed: Recognizing Faces and Feelings to Improve Communication and Emotional Life*. New York: Times Books

[B7] Ekman P. Friesen W. V. (1978). *Facial Action Coding System: A Technique for the Measurement of Facial Action*. Palo Alto, CA: Consulting Psychologists Press

[B8] Gagnon M. Gosselin P. Hudon-ven der Buhs I. Larocque K Milliard K. (2010). Children’s recognition and discrimination of fear and disgust facial expressions. *J. Nonverbal Behav.* 34 27–42 10.1007/s10919-009-0076-z

[B9] Gosselin P. (1995). The development of the recognition of facial expressions of emotion in children. *Can. J. Behav. Sci.* 27 107–119 10.1037/008-400X.27.1.107

[B10] Gosselin P. Larocque C. (2000). Facial morphology and children’s recognition of facial expressions of emotions: a comparison between Asian and Caucasian faces. *J. Genet. Psychol.* 161 346–358 10.1080/00221320009596717 10971913

[B11] Gosselin P. Roberge P Lavallée M. C. (1995). The development of the recognition of facial emotional expressions comprised in the human repertoire. *Enfance* 4 379–396 10.3406/enfan.1995.2144

[B12] Harrigan J. A. (1984). The effects of task order on children’s identification of facial expressions. *Motiv. Emot.* 8 157–169 10.1007/BF00993071

[B13] Izard C. E. (1991). *The Psychology of Emotion*. New York: Plenum 10.1007/978-1-4899-0615-1

[B14] Lété B. Sprenger-Charolles L Colé P. (2004). Manulex: a grade-level lexical database from French elementary-school readers. *Behav. Res. Methods Instrum. Comput.* 36 156–166 10.3758/BF03195560 15190710

[B15] MacWhinney B. (2000). *The CHILDES Project: Tools for Analyzing Talk*, 3rd Edn. Mahwah, NJ: Lawrence Erlbaum Associates

[B16] Markham R. Adams K. (1992). The effect of type of task on children’s identification of facial expressions. *J. Nonverbal Behav.* 16 21–39 10.1007/BF00986877

[B17] Ridgeway D. Waters E. Kuczaj S. A. (1985). Acquisition of emotion-descriptive language: receptive and productive vocabulary norms for ages 18 months to 6 years. *Dev. Psychol.* 21 901–908 10.1037/0012-1649.21.5.901

[B18] Russell J. A. (1994). Is there universal recognition of emotion from facial expression? A review of the cross-cultural studies. *Psychol. Bull.* 115 102–141 10.1037/0033-2909.115.1.102 8202574

[B19] Sander D. Scherer K. R. (2009). *Traité de psychologie des émotions*. Paris: Dunod

[B20] Shweder R. A. Haidt J. Horton R. Joseph C. (2008). “The cultural psychology of the emotion: Ancient and renewed,” in *Handbook of Emotions* eds Lewis M. Haviland-Jones J. M. Feldman Barrett L. (New York: The Guildford Press) 409–427

[B21] Tremblay C. Kirouac G Doré F. (1987). The recognition of adults’ and children’s facial expressions of emotions. *J. Psychol.* 12 341–350 10.1080/00223980.1987.9712674

[B22] Vicari S. Snitzer Reilly J. Pasqualetti P. Vizzotto A Caltagirone C. (2000). Recognition of facial expressions of emotions in school-age children: the intersection of perceptual and semantic categories. *Acta Paediatr.* 89 836–845 10.1111/j.1651-2227.2000.tb00392.x 10943968

[B23] Wellman H. M. Harris P. L. Banerjee M. Sinclair A. (1995). Early understanding of emotion: evidence from natural language. *Cogn. Emot.* 9 117–149 10.1080/02699939508409005

[B24] Widen S. C. Russell J. A. (2003). A closer look at preschoolers freely produced labels for facial expressions. *Dev. Psychol.* 39 114–128 10.1037/0012-1649.39.1.114 12518813

[B25] Widen S. C. Russell J. A. (2008). Children’s and adults’ understanding of the disgust face. *Cogn. Emot.* 22 1513–1541 10.1080/02699930801906744

[B26] Widen S. C. Russell J. A. (2010). The disgust face conveys anger to children. *Emotion* 10 455–466 10.1037/a0019151 20677863

[B27] Widen S. C. Russell J. A. (2013). Children’s recognition of disgust in others. *Psychol. Bull.* 139 271–299 10.1037/a0031640 23458434

[B28] Zuckerman M. Przewuzman S. J. (1979). Decoding and encoding facial expressions in preschool-age children. *Environ. Psychol. Nonverbal Psychol.* 3 147–163 10.1007/BF01142589

